# Cortisol Diurnal Rhythm and Stress Reactivity in Male Adolescents with Early-Onset or Adolescence-Onset Conduct Disorder

**DOI:** 10.1016/j.biopsych.2008.05.022

**Published:** 2008-10-01

**Authors:** Graeme Fairchild, Stephanie H.M. van Goozen, Sarah J. Stollery, Jamie Brown, Julian Gardiner, Joe Herbert, Ian M. Goodyer

**Affiliations:** a Developmental Psychiatry Section, Department of Psychiatry, Cambridge University, United Kingdom; b School of Psychology, Cardiff University, United Kingdom; c Department of Physiology, Development and Neuroscience, Cambridge University, United Kingdom

**Keywords:** Antisocial behavior, conduct disorder, cortisol, cortisol awakening response, HPA axis, stress reactivity

## Abstract

**Background:**

Previous studies have reported lower basal cortisol levels and reduced cortisol responses to stress in children and adolescents with conduct disorder (CD). It is not known whether these findings are specific to early-onset CD. This study investigated basal and stress-induced cortisol secretion in male participants with early-onset and adolescence-onset forms of CD.

**Methods:**

Forty-two participants with early-onset CD, 28 with adolescence-onset CD, and 95 control subjects participated in the study. They collected saliva across the day to assess their cortisol awakening response and diurnal rhythm. Subsequently, salivary cortisol was measured before, during, and after a psychosocial stress procedure designed to elicit frustration. Cardiovascular activity and subjective mood states were also assessed during stress exposure.

**Results:**

There were no group differences in morning cortisol levels or the size of the cortisol awakening response. Basal cortisol levels in the evening and at 11 am during the laboratory visit were higher in both CD subgroups relative to control subjects. In contrast, cortisol and cardiovascular responses to psychosocial stress were reduced in both CD subgroups compared with control subjects. All groups reported similar increases in negative mood states during stress.

**Conclusions:**

Our findings suggest that group differences in cortisol secretion are most pronounced during stress exposure, when participants with CD show cortisol hyporeactivity compared with control subjects. There was no evidence for reduced basal cortisol secretion in participants with CD, but rather increased secretion at specific time points. The results do not support developmentally sensitive differences in cortisol secretion between CD subtypes.

Previous psychophysiological research on conduct disorder (CD) has examined whether deficits in the stress response account for the emergence of antisocial behaviors. Both the hypothalamic-pituitary-adrenal (HPA) axis and the autonomic nervous system have been investigated. Previous studies have reported reduced cortisol levels in individuals with CD or oppositional defiant disorder (ODD) (1–3) or an inverse relationship between cortisol levels and CD symptoms (4,5). Longitudinal studies have shown that lower basal cortisol predicts aggressive behavior or is a marker for persistent aggression (6,7). In contrast, several studies failed to demonstrate any relationship between basal cortisol levels and a CD/ODD diagnosis (8–10), and one reported increased cortisol levels in CD (11; see ref. 12 for a review). The heterogeneity of these results may be due to the use of different samples (clinic-referred vs. population-based), informants (self-report, parental or teacher report), or measures (urinary, plasma, or salivary cortisol). In addition, some studies failed to control for time of day, which is problematic given the marked diurnal rhythm of cortisol (13). Single-point saliva or blood sampling, as occurred in many studies, is not optimal because cortisol levels are responsive to stress. This study improved on previous research by characterizing the diurnal profile of cortisol secretion in CD, including the cortisol awakening response (CAR) in the hour after waking (14,15).

In addition to basal cortisol, we investigated cortisol secretion during psychosocial stress. Cortisol responses to stress are reduced in children with ODD (10) and juvenile delinquents with an ODD/CD diagnosis (16). This blunted cortisol response appears relatively specific to ODD/CD (17) and has predictive value in distinguishing between groups of children who will respond favorably or otherwise to psychological interventions (18).

As well as cortisol secretion, basal heart rate is reported to be reduced in children and adolescents with severe antisocial behavior (19). Longitudinal studies have shown that low heart rate predicts aggression and antisocial behavior (20,21), whereas high heart rate may be a protective factor in those at risk of developing criminal behavior (22). Differences in cardiovascular reactivity to stress are less consistent, with some studies reporting a blunted response (10,16) but others an enhanced cardiovascular response to stress (3). We examined cardiovascular activity under basal conditions and during stress to clarify these issues.

Finally, this study distinguished between the early-onset and adolescence-onset subtypes of CD. It has been suggested that individuals with early-onset CD (EO-CD) show neuropsychological impairments. In contrast, adolescence-onset CD (AO-CD) is considered to arise primarily because of social modeling of deviant peers (23). We investigated whether this hypothetical distinction between CD subtypes would extend to differences in patterns of cortisol secretion or cardiovascular activity under basal conditions or during stress. This issue is theoretically and clinically significant given the distinction made in the DSM-IV (24) between childhood-onset and adolescence-onset forms of CD and data indicating a positive relationship between cortisol reactivity and response to treatment (18). A failure to distinguish between CD subtypes may underlie some previously inconsistent findings in this area.

Our primary hypothesis was that community-based adolescents with EO-CD would show reduced basal cortisol levels (including a blunted CAR), as well as cortisol and cardiovascular hyporeactivity during psychosocial stress, relative to control subjects. A secondary objective was to provide similar data in participants with AO-CD to allow comparison with EO-CD and control participants.

## Methods and Materials

### Participants

Male adolescents aged 14–18 years were recruited from schools and colleges, pupil referral units, and the Cambridge Youth Offending Service. We recruited 95 control subjects (no history of CD/ODD and no current psychiatric illness) and 70 index cases, of whom 42 received an EO-CD diagnosis and 28 received an AO-CD diagnosis. All participants gave written informed consent, and the study was approved by the local research ethics committee.

Exclusion criteria included IQ under 75 as assessed using the Vocabulary and Block Design subtests of the Wechsler Abbreviated Scale of Intelligence (WASI) (25), presence of pervasive developmental disorder or chronic physical illness, and use of steroid medication.

Participants were assessed for CD, ODD, attention-deficit/hyperactivity disorder (ADHD), major depressive disorder (MDD), generalized anxiety disorder (GAD), obsessive-compulsive disorder (OCD), and posttraumatic stress disorder using the Schedule for Affective Disorders and Schizophrenia for School-Age Children—Present and Lifetime Version (K-SADS-PL) (26), which reflects DSM-IV criteria (24). Separate diagnostic interviews were carried out with the participants themselves and their main caregivers.

Participants were allocated to the EO-CD group if they or their caregivers reported at least one CD symptom and functional impairment was present before age 10 years (24), or if they met full criteria for ODD before age 10 and developed CD after age 10. If onset occurred after age 10, an AO-CD diagnosis was given. Inclusion in the study was based on lifetime diagnoses of CD, although 94.3% of index cases had a current CD diagnosis.

Anxiety symptoms were assessed using the Revised Children's Manifest Anxiety Scale (27). Psychopathic traits were measured using the Youth Psychopathic Inventory (28), and socioeconomic status (SES) was estimated using the Standard Occupational Classification 2000 guidelines. Substance use was assessed using the Adolescent Alcohol and Drug Involvement Scale (29).

Eleven participants with EO-CD and five with AO-CD had current comorbid ADHD (all had been medication-free for more than 6 months). One AO-CD and five EO-CD participants had current comorbid MDD. Four control, seven EO-CD, and five AO-CD participants had past MDD. Finally, one AO-CD participant had comorbid GAD, and one EO-CD participant had comorbid OCD.

### Procedure for Saliva Collection Under Basal Conditions

Participants collected saliva in polyethylene vials using a “passive drool” method (i.e., without aids to salivation) at four time points across the day: immediately after waking (Sample 1), +30 and +60 min after waking (Samples 2 and 3), and at 9 pm (Sample 4) for 3 consecutive weekdays.

Participants were asked to write the sampling times in a “spit diary.” They were asked to rinse their mouths with water and then wait approximately 1 min before producing each sample and to avoid smoking, eating, drinking caffeinated or alcoholic drinks, taking recreational drugs, engaging in vigorous exercise, or brushing their teeth until the first three samples had been collected or in the 2 hours preceding Sample 4. Compliance was monitored using the spit diary. Participants were informed that the accuracy of sampling could be determined from the lab results, in an attempt to improve compliance (30). All samples were frozen after collection and brought into the lab in a semifrozen state. They were stored at −20°C until assay.

### Psychosocial Stress Induction Procedure

Participants arrived at the department in the morning and completed a battery of questionnaires and neuropsychological tests and the WASI. Approximately 60–75 min after lunch, they were informed that they would be taking part in a competition with an opponent of a similar age with a cash prize for the winner. This procedure is described elsewhere (3); briefly, it involves inducing frustration and provocation between the participant and a prerecorded video opponent.

The competition began between 1 and 2 pm with a task involving confrontation, the Prisoner's Dilemma Game (PDG), in which the opponent always failed to cooperate and sent antagonistic messages. Frustration was induced by having the participant perform a difficult, computer-based manual precision task (MPT) under time pressure while the video opponent and experimenter watched. By design, all participants failed to achieve their target score and received negative evaluations of their performance from the opponent. Following these tasks, participants completed further challenging cognitive tasks aimed at increasing performance uncertainty. Finally, they watched their opponent play the MPT and could remotely disrupt the opponent's performance. At the end of the session (between 3 and 4 pm), participants were told they had won the competition.

An additional group of control subjects (*n* = 12) took part in a nonstressful version of the afternoon session (which involved watching benign video clips and filling in questionnaires) to examine the efficacy of the stressor. Saliva was collected at similar intervals.

### Procedure for Saliva Collection Before and During the Psychosocial Stressor

Saliva was again collected by passive drool. If the participant was experiencing difficulty spitting, sugar- and flavor-free chewing gum (Trident Sugar Free Neutral) was provided to assist salivation. Two baseline and five stress samples were collected at the following time points: 1) at 11 am during the morning session (baseline, prelunch); 2) before the competition (baseline, −5 min); 3) following performance of the PDG and MPT and negative social evaluation (+35 min after stress onset) and at four subsequent time points at 25-min intervals (+60, +85, +110, and +135 min after stress onset).

### Cortisol Analysis

Cortisol was measured by enzyme-linked immunosorbent assay on 20-μL duplicates of unextracted saliva samples (antibody Cambio, Cambridge, United Kingdom). The intraassay and interassay coefficients of variation were 4.37% and 7.62%, respectively. Results are reported in nmol/L.

### Procedure for Heart Rate Measurement

Participants were seated throughout the session and asked to remain as still as possible. Heart rate (HR) was measured using the ECG100C amplifier unit (BIOPAC Systems, Goleta, California) and disposable cardiac electrodes (Vermed, Bellows Falls, Vermont), which were fixed to the wrists of the participants. Data were recorded at a rate of 100 Hz using the MP150 system (BIOPAC Systems). HR was recorded for 5 min while the participant was at rest to yield baseline (−5 Min) values for HR and continuously during the stress procedure. Data were analyzed offline using AcqKnowledge 3.7.2 (BIOPAC Systems).

### Recording of Psychological States

Participants rated their feelings eight times using an adaptation of a clinical self-rating scale (31). The scale contained 11 items (happy/gloomy, well/sick, cheerful/not cheerful, good/bad, liked/not liked, satisfied/not satisfied, worried/not worried, embarrassed/not embarrassed, ashamed/not ashamed, afraid/not afraid, and angry/not angry), which participants rated using 9-point ordinal scales. They also rated their feelings of control and confidence about winning the competition. Subjective ratings occurred at similar times as saliva collection, apart from the second rating, which was completed after the PDG and the fifth and sixth ratings (before and after the opponent played the MPT).

### Data Analysis

**Figure 1:**
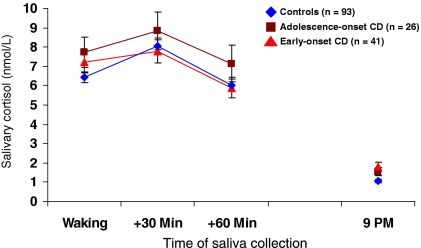
Mean (± SEM) salivary cortisol levels under basal conditions by group, showing cortisol levels at waking, +30 min after waking, +60 min after waking, and at approximately 9 pm, averaged across 3 consecutive week days. All groups exhibited a marked cortisol diurnal rhythm and a cortisol awakening response in the first hour after waking. Evening cortisol levels were significantly higher in the CD subgroups relative to controls. CD, conduct disorder.

**Figure 2:**
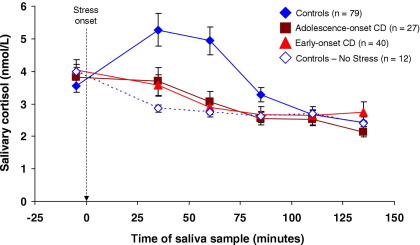
Mean (± SEM) salivary cortisol levels at seven time points, by experimental group. Seventy-nine control subjects and all adolescence-onset and early-onset CD cases were exposed to psychosocial stress. Under stressful conditions, the elevation in cortisol levels between baseline (−5 min) and +35 min in control subjects was markedly reduced in participants from both CD subgroups. The dashed arrow shows onset of the psychosocial stressor, and all times are shown relative to stressor onset. The dashed line and open diamond symbols show data from 12 control subjects that were not exposed to stress for comparison purposes. CD, conduct disorder.

**Figure 3:**
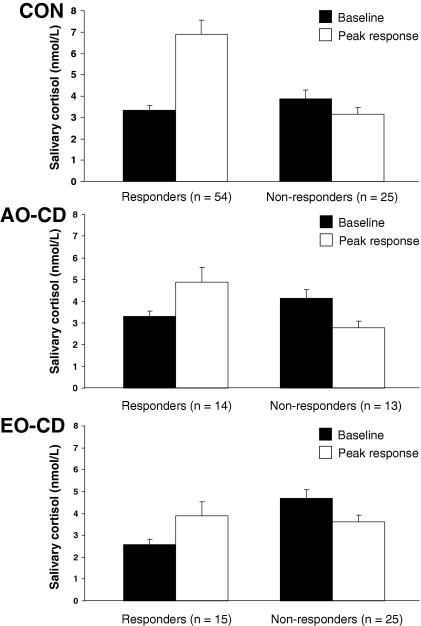
Proportion of cortisol responders and nonresponders in each experimental group, using the criterion of an increase in cortisol levels of 15% or above, relative to baseline (−5 min). The black bars show cortisol levels at baseline in each group, and the white bars show the direction and mean peak magnitude of change (± SEM) in salivary cortisol levels following exposure to psychosocial stress. CON, control subjects; AO-CD, adolescence-onset conduct disorder; EO-CD, early-onset conduct disorder.

The raw cortisol values were positively skewed and normalized using a log transformation. However, Figures 1–3 show absolute cortisol values so as to be physiologically meaningful.

Chi-square or one-way analysis of variance (ANOVA) tests were used to assess group differences in demographic variables, as appropriate.

For basal cortisol analyses, mixed-effects models (32) were fitted to the data using restricted maximum likelihood estimates in R 2.2.1 (http://www.r-project.org/). The CAR was quantified using the area under the curve (AUC_I_) value for cortisol increase relative to the waking value (33).

To assess group differences in cortisol, HR, and self-reported affect changes during stress, repeated-measures ANOVAs were performed with group as a between-subjects factor and time as a within-subjects factor. When the assumption of sphericity was violated, degrees of freedom were corrected using Greenhouse-Geisser procedures (34). Tukey tests were used for post hoc group comparisons.

To quantify cortisol responsiveness to psychosocial stress, AUC_I_ values were calculated for the cortisol increase across samples 2–7, with reference to the baseline value before stressor onset (−5 min) (33). A one-way ANOVA was used for group comparisons.

Effect sizes are reported as partial eta-squared (η_p_^2^; small ≥ .01, medium ≥ .06, large ≥ .14) for repeated-measures ANOVA or regression analyses, or in terms of Cohen's *d* (small ≥ .2, medium ≥ .5, large ≥ .8) (35) for all other group comparisons. Analyses were performed using SPSS 11.5 (SPSS, Chicago, Illinois), S-Plus 6.2 (Insightful, Seattle, Washington), and R 2.2.1.

## Results

### Demographics

See Table 1 for demographic and personality characteristics of each group. The EO-CD group was of lower SES than the control or AO-CD groups (both *p*s < .001). There were more nonwhite participants in the AO-CD group than the EO-CD group (*p* < .01), although neither group differed from control subjects in ethnicity.

### Basal Cortisol

Two control subjects and one EO-CD participant failed to collect saliva samples before their laboratory visit. Data from two other AO-CD participants were excluded because of noncompliance. Table 2 shows mean saliva collection times for each group. The groups differed in waking time, with control subjects waking more than 1 hour earlier than participants from either CD subgroup on each day of collection. However, they reported similar levels of compliance with the collection protocol (i.e., in terms of the intervals between respective samples) after waking. Sampling time did not differ between groups in the evening on Days 1 and 2 but was significantly later in the EO-CD group relative to control subjects on Day 3 (*p* < .05).

Figure 1 shows absolute cortisol levels by group averaged across Days 1–3. The mixed-effects model that best fit the data had separate group means but a common diurnal slope (because the group difference in slope values was only marginally significant, *p =* .053). The slope value across all participants was −.145 [*t*(1241) = −29.6, *p* < .0001], demonstrating a highly significant diurnal decline in cortisol levels. Intercept (estimated cortisol centered at 12 noon) values differed between groups [χ^2^(2) = 9.93, *p =* .007], with higher cortisol levels observed in both CD subgroups than control subjects. Individual group comparisons showed a significant difference between control and EO-CD participants (*p* < .05). Further results of the mixed-effects analyses, including subject-specific and day-specific variance components, and intraclass correlation coefficients are provided in Table 3. The fact that intraclass correlation coefficient values were relatively high suggests that much of the variance in cortisol measurements observed is attributable to robust between-individual differences.

There was no group effect on cortisol levels at any of the am sampling times (Sample 1, *p =* .81; Sample 2, *p =* .69; Sample 3, *p =* .61) or on the AUC_I_ value for the CAR [*F*(2,398) = 1.38, *p =* .25]. There was, however, a significant group difference in pm cortisol [χ^2^(1) = 15.12, *p* < .001]. Cortisol levels were higher in both CD subgroups relative to control subjects (post hoc: control subjects vs. AO-CD, *p* < .05; control subjects vs. EO-CD, *p* < .001).

### Cortisol Levels Before and During Stress

#### Effect of Frustration/Provocation in Control Subjects

Four control, one AO-CD and two EO-CD subjects did not participate in the stress experiment or collect saliva under nonstress conditions. Cortisol levels declined relative to baseline in control subjects unexposed to stress, whereas the frustration/provocation procedure elicited a robust increase in cortisol secretion: group × time interaction [F(2.20,193.80) = 4.28, *p* < .05, η_p_^2^ = .05]; Figure 2).

#### Group Effects on Cortisol at Baseline and During Stressor

Mean (± SEM) cortisol levels at 11 am on the testing day were higher in both CD subgroups than in control subjects [control subjects: 3.02 (± .23) nmol/L, AO-CD: 4.13 (± .40) nmol/L, EO-CD: 3.94 (± .32) nmol/L; *F*(2,145) = 7.99, *p* < .001, η_p_^2^ = .10]; post hoc comparison showed that both CD subgroups differed from control subjects (*p* < .005; *d* = .69 and .62 for AO-CD and EO-CD, respectively). Cortisol levels did not differ between groups at the pretest baseline (−5 min; *p =* .37).

Figure 2 shows that, following stress onset, cortisol levels diverged in the groups: control subjects exposed to stress showed a clear increase, whereas both CD subgroups showed declines over the same period. This decline was similar to that observed in control subjects not exposed to stress. Repeated-measures ANOVA using cortisol levels at baseline through to +110 min as the dependent variables showed an effect of time [*F*(2.82,402.80) = 33.02, *p* < .001, η_p_^2^ = .19], but no effect of group [*F*(2,143) = 1.76, *p =* .18]. Critically, there was a significant group × time interaction [*F*(5.63,402.80) = 5.34, *p* < .001, η_p_^2^ = .07].

Supporting this interaction effect, comparison of AUC_I_ values for cortisol reactivity revealed a main effect of group [*F*(2,143) = 10.35, *p* < .001, η_p_^2^ = .13]; post hoc comparisons showed that both CD groups had lower AUC_I_ values than control subjects (*p* < .01, *d* = .68 for control subjects vs. AO-CD; *p* < .001, *d* = .75 for control subjects vs. EO-CD). The CD groups did not differ from each other.

We also investigated variation in cortisol reactivity within each group by dividing participants into responders and nonresponders, using the criterion of an increase in cortisol levels of 15% or more between baseline and either the +35-min or +60-min time points (36). By this criterion, 54 control participants (68%), 14 AO-CD participants (52%), and 15 EO-CD participants (38%) qualified as cortisol responders (Figure 3). A comparison of groups in proportion of responders and nonresponders revealed a significant difference between control and EO-CD groups [χ^2^(1) = 10.4, *p* < .001]; the AO-CD group did not differ significantly from either of the other groups.

We then examined whether the magnitude of the cortisol response in those participants deemed responders differed by group. Control responders showed a mean (± SEM) cortisol increase of 3.57 (± .49) nmol/L, whereas AO-CD and EO-CD responders showed mean increases of 1.59 (± .36) nmol/L and 1.31 (± .25) nmol/L, respectively [*F*(2,82) = 4.83, *p* < .01, η_p_^2^ = .11; post hoc: control subjects vs. EO-CD *p* < .05; control subjects vs. AO-CD *p =* .08; EO-CD vs. AO-CD *p =* .97].

### Heart Rate

**Figure 4:**
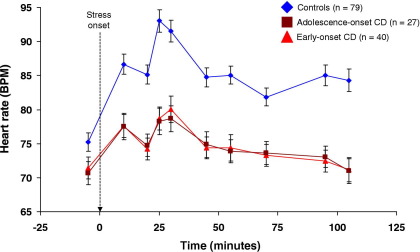
Mean (± SEM) heart rate, expressed in beats per minute (BPM), across the 10 tasks that formed the psychosocial stressor, by group. Heart rate levels did not differ significantly at baseline, but cardiovascular responses to stress were markedly attenuated in both CD subgroups relative to control subjects. The dashed arrow shows onset of the psychosocial stressor, and all times are shown relative to its onset. CD, conduct disorder.

Figure 4 shows that stress-induced changes in heart rate (HR) were attenuated in both CD subgroups, relative to control subjects. Although HR was lower in both CD subgroups at baseline (−5 Min), the group difference was not statistically significant [*F*(2,143) = 2.54, *p =* .08].

Repeated-measures ANOVA revealed main effects of time [*F*(5.79,827.93) = 49.37, *p* < .001, η_p_^2^ = .26], and group [*F*(2,143) = 17.06, *p* < .001, η_p_^2^ = .19], and a significant group × time interaction [*F*(11.58,827.93) = 7.37, *p* < .001, η_p_^2^ = .09]. The interaction was driven by control subjects showing greater increases between prestress and peak HR values than both CD subgroups (mean beat per minute increase for control subjects: 17.8; AO-CD participants: 7.8; EO-CD participants: 7.3; post hoc on group effect: control subjects vs. AO-CD, *p* < .001, η_p_^2^ = .15; control subjects vs. EO-CD, *p* < .001, η_p_^2^ = .17; AO-CD vs. EO-CD, *p =* .99).

### Subjective Changes

**Figure 5:**
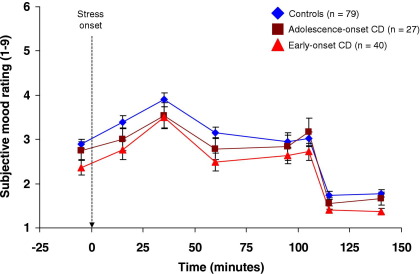
Mean (± SEM) self-reported changes in subjective feelings by group, as assessed using Von Zerssen scales. The scales in question involve rating subjective feelings on a continuum between 1 and 9, with presence of positive emotion or absence of negative emotion indicated by low numbers, and absence of positive emotion or presence of negative emotion indicated by high numbers. All groups showed an increase in negative feelings and a reduction in positive feelings following the onset of psychosocial stress, and the changes appeared to take a parallel course in each experimental group. The dashed arrow shows onset of the psychosocial stressor, and all times are shown relative to stress onset. CD, conduct disorder.

Data from the first six reports of subjective states completed under psychosocial stress were analyzed (ratings 7 and 8 were obtained under nonstress conditions). Figure 5 indicates that all groups showed parallel increases in negative feelings and decreases in positive feelings during stress.

There was a main effect of time on the mean negative affect score summed across all 11 emotions [*F*(4.16,595.52) = 21.42, *p* < .001, η_p_^2^ = .13] and a trend effect of group [*F*(2,143) = 2.74, *p =* .07], but there was no group × time interaction (*p =* .43).

We analyzed individual items on the rating scales to examine further whether stress affected the groups differentially. Seven items showed significant changes (toward negative affect), as shown by main effects of time, but no effects of group or group × time interactions. Group effects were seen for feelings of fear, worry, embarrassment, and shame, with post hoc tests showing lower levels of these emotions in EO-CD participants relative to control subjects. However, the only significant group × time interaction was for feelings of worry [*F*(2,143) = 3.98, *p =* .02, η_p_^2^ = .05]. EO-CD participants reported increases, whereas control subjects and AO-CD participants reported decreases, in worried feelings during the stressor.

There were effects of time on controllability (*p* < .001) and confidence about winning (*p* < .001), with participants feeling less in control (all *p* < .01) and less confident (*p* < .05) at all time points during the stressor relative to baseline. However, there were no group effects on these variables or group × time interactions.

### Possible Confounds

Because of previous reports of cortisol hyporeactivity in adult smokers (37,38), we examined the impact of smoking. The proportion of participants in each group showing a cortisol response was similar in smokers and nonsmokers (control subjects: 58% vs. 69%; AO-CD: 48% vs. 52%; EO-CD: 36% vs. 60%; Fisher's exact tests all *p*s > .1). Comparison of AUC_I_ values for cortisol response revealed no differences between smokers and nonsmokers in any group. There were no other significant covariates in the analysis of AUC_I_ values for cortisol reactivity.

Estimated IQ was a significant covariate in the analysis of HR responses to stress (*p* < .01), although the group effect remained significant after covarying for IQ (*p* < .005). None of the other demographic variables were significant covariates.

## Discussion

The findings on basal cortisol secretion demonstrate that the magnitude of the CAR was similar across groups. In contrast, both CD subgroups showed increased mean cortisol levels compared with control subjects, largely because of higher evening levels. All groups showed significant declines in cortisol levels across the day, and cortisol slope values did not differ significantly between groups. Adolescents with EO-CD exhibited reduced cortisol and cardiovascular responses to psychosocial stress relative to control subjects. Furthermore, the mean cortisol response to stress was smaller in EO-CD participants compared with control subjects, even if only those participants who exhibited a measurable cortisol response to stress were considered. Participants with AO-CD displayed a strikingly similar pattern of cortisol and cardiovascular hyporeactivity to that observed in those with EO-CD. There was also a trend toward lower cortisol reactivity in AO-CD responders (despite the loss of power incurred by considering only those with measurable responses). The size of the peak cortisol response observed was also less than half that seen in responders from the control group. As such, there was no evidence for differences in basal cortisol secretion and physiologic reactivity to stress between AO-CD and EO-CD participants.

Differences in emotional responses did not explain the cortisol and cardiovascular hyporeactivity findings because increases in self-reported negative affect during stress followed a similar course across groups. This discrepancy between subjective and physiologic changes suggests poorer coordination between emotional and physiologic arousal in both CD subtypes (10,16).

Our basal cortisol findings, showing an intact CAR and cortisol diurnal rhythm but elevated 11 am and evening cortisol levels in both forms of CD, are consistent with previous results in population-based samples (11,39). They are, however, at variance with studies showing lower cortisol levels in clinic-referred CD samples (2,6). The latter studies obtained single-point cortisol measurements or may have been confounded with acute stress due to venipuncture. An earlier study reported that prepubertal boys with disruptive behavior disorders had similar CAR magnitudes compared with control subjects, using the AUC_I_ value for cortisol (40). However, in contrast to our results, that study observed reduced absolute cortisol secretion over the first hour after waking in CD/ODD participants relative to control subjects.

Our findings of cortisol and cardiovascular hyporeactivity during stress in CD are consistent with previous work in clinic-referred children with ODD (3,10) and delinquent prepubertal adolescents (16). They are also in agreement with results in high-risk children of fathers with substance dependence (41,42). These data indicate that, unlike reduced basal cortisol, cortisol and cardiovascular hyporeactivity are associated with CD in both clinic-referred and population-based samples. This has clinical implications because cortisol hyporeactivity during stress is associated with poor treatment outcome (18), and some treatments for CD encourage patients to become aware of their physiologic reactions as triggers for anger states. Remaining questions relate to the physiologic origin of the cortisol hyporeactivity observed in CD and its implications for psychological functioning. Future studies should examine whether cortisol hyporeactivity precedes CD onset using longitudinal designs.

Our findings of blunted cortisol and cardiovascular responses to stress in AO-CD and EO-CD participants relative to control subjects contradicts the developmental taxonomic theory, which implies that such neurobiological differences should be unique to EO-CD. Physiologic hyporeactivity during stress could reflect a latent trait that increases vulnerability to CD, whereas age of CD onset may be moderated by psychosocial factors (e.g., differences in parental supervision, exposure to antisocial models). Alternatively, it may be unnecessary to invoke a latent trait in either subgroup: rather, both CD subgroups may have experienced increased social adversity during development (e.g., maltreatment), or, because of heightened risk-taking behaviors, they may place themselves in stressful situations more frequently than other adolescents (leading to habituation to stressors).

Two limitations are noted. First, aside from spit diaries, no measures were in place to ensure compliance with the saliva collection protocol. This is problematic because poor adherence may be expected in adolescents with CD and may have resulted in elevated evening cortisol in the CD subgroups. The robust cortisol response to awakening and diurnal rhythm observed in all three groups nevertheless suggests that, in most cases, participants followed the collection protocol with acceptable accuracy. Furthermore, increased basal cortisol levels were observed in both CD subgroups at 11 am when under experimental supervision.

Second, although we obtained information from multiple informants and enquired about the age of onset of each CD and ODD symptom, the study relied on retrospective accounts. As such, the findings require replication in a longitudinal design, including follow-up to the point of remission.

In summary, this study demonstrated that cortisol and cardiovascular responses to an ecologically valid psychosocial stressor were reduced in adolescents with both early-onset and adolescence-onset CD. These findings were not explained by differences in subjective responses to the stressor, suggesting a discrepancy between mood changes and physiologic reactivity in CD. The basal cortisol data showed a normal diurnal rhythm and CAR in participants with CD, suggesting intact HPA axis function. Finally, contrary to predictions, we observed elevated 11 am and evening cortisol in both CD subgroups.

## Figures and Tables

**Table 1:** Participant Characteristics

	CON (*n* = 95)	AO-CD (*n* = 28)	EO-CD (*n* = 42)	Group Effect (*p* Value)	Significant Post Hoc Comparisons
Age (years)	15.69 ± .85	15.61 ± .86	15.79 ± .81	.69	
Estimated IQ	106.72 ± 12.13	99.29 ± 11.42	92.76 ± 10.63	<.001	CON > AO, EO
Anxiety	7.24 ± 4.79	9.64 ± 5.84	10.18 ± 5.03	.004	CON < EO
Psychopathic Traits	2.08 ± .30	2.34 ± .25	2.47 ± .34	<.001	CON < AO, EO
CD Symptoms	.26 ± .65	6.43 ± 1.99	8.29 ± 3.05	<.001	CON < AO < EO
SES					
Low	11 (11.6%)	6 (21.4%)	17 (40.5%)	<.001	
Middle	14 (14.7%)	9 (32.1%)	12 (28.6%)		
High	64 (67.4%)	12 (42.8%)	7 (16.7%)		
Ethnicity					
Caucasian	86 (90.5%)	22 (78.6%)	42 (100%)	.009	
Mixed-Race	4 (4.2%)	4 (14.3%)			
Asian/Black	5 (5.3%)	2 (7.1%)			
Habitual use of Tobacco	13 (13.7%)	21 (75.0%)	31 (73.8%)	<.001	CON < AO, EO
Regular use of					
Alcohol	3 (3.2%)	3 (10.7%)	10 (23.8%)	<.001	CON < EO
Cannabis	8 (8.4%)	14 (50.0%)	18 (42.9%)	<.001	CON < EO

Data are presented as means ± SD or number and percentage (in parenthesis) in each group. SES information was unavailable for 6 control, 1 AO-CD, and 6 EO-CD participants. CON, control subjects; AO-CD, adolescence-onset conduct disorder; EO-CD, early-onset conduct disorder; SES, socioeconomic status.

**Table 2:** Self-Reported Saliva Collection Times for the Control, AO-CD, and EO-CD Groups, Respectively, Showing Group Means (± SD)

Day	Sample Time	Control	AO-CD	EO-CD
1	Awakening	07:56 ± 01:22	09:47 ± 02:15	09:33 ± 01:27
	+30 min	08:27 ± 01:22	10:17 ± 02:15	10:05 ± 01:28
	+60 min	08:59 ± 01:22	10:50 ± 02:16	10:41 ± 01:28
	9 pm	21:06 ± 00:43	21:19 ± 00:37	21:23 ± 00:42
2	Awakening	08:19 ± 02:12	09:15 ± 01:38	09:43 ± 01:44
	+30 min	08:50 ± 02:12	09:49 ± 01:38	10:08 ± 01:38
	+60 min	09:23 ± 02:12	10:26 ± 01:43	10:49 ± 01:45
	9 pm	21:08 ± 00:39	21:30 ± 00:53	21:43 ± 01:13
3	Awakening	08:20 ± 01:50	09:54 ± 01:50	09:38 ± 01:43
	+30 min	08:49 ± 01:49	10:29 ± 02:01	10:10 ± 01:42
	+60 min	09:20 ± 01:49	10:54 ± 01:51	10:41 ± 01:43
	9 pm	21:12 ± 00:35	21:16 ± 00:36	21:38 ± 00:58

AO-CD, adolescence-onset conduct disorder; EO-CD, early-onset conduct disorder.

**Table 3:** Models of Cortisol's Diurnal Rhythm with Individual Differences in Mean Levels in the Three Groups, and Day-Level Differences Across Groups (Cortisol Expressed as Natural Logarithm [nmol/L])

	Value	SEM
Fixed Effects		
Intercept^a^ (CON group)	.147	.057
Intercept^a^ (AO-CD group)	.420	.111
Intercept^a^ (EO-CD group)	.439	.087
Overall Slope	−.145	.005
Variance Components		
Subject-Specific Means	.242	
Day-Specific Means	.058	
Residual Variance	.294	
ICC Estimates		
ICC (Between Days)	.407	
ICC (Within Days)	.505	

AO-CD, adolescence-onset conduct disorder; CON, control subjects; EO-CD, early-onset conduct disorder; ICC, intraclass correlation coefficient (ranges from 0 to 1, with higher values indicating a greater degree of correlation among measurements from the same individual). Data are presented as means ± SEM for each group.

a Estimated mean log cortisol centered on 12 noon.
